# Beyond Brooding on Oncometabolic Havoc in IDH-Mutant Gliomas and AML: Current and Future Therapeutic Strategies

**DOI:** 10.3390/cancers10020049

**Published:** 2018-02-11

**Authors:** Hanumantha Rao Madala, Surendra R. Punganuru, Viswanath Arutla, Subhasis Misra, T. J. Thomas, Kalkunte S. Srivenugopal

**Affiliations:** 1 Department of Biomedical Sciences, School of Pharmacy, Texas Tech University Health Sciences Center, Amarillo, TX 79106, USA; hanumantharao.madala@ttuhsc.edu (H.R.M.); Surendra.r.punganuru@ttuhsc.edu (S.R.P.); Viswanath.Arutla@ttuhsc.edu (V.A.); 2 Surgical Oncology, Brandon Regional Hospital, Brandon, FL 33511, USA; Subhasis.Misra@hcahealthcare.com; 3 Department of Medicine, Rutgers Robert Wood Johnson Medical School and Rutgers Cancer Institute of New Jersey, Rutgers, The State University of New Jersey, Piscataway, NJ 08854, USA; thomastj@rwjms.rutgers.edu

**Keywords:** IDH mutations, D-2 hydroxyglutarate, epigenetic effects, hydroxy-methyl cytosine, histone methylations, glioblastoma, hematopoietic cancers, α-KG-dependent dioxygenases

## Abstract

Isocitrate dehydrogenases 1 and 2 (IDH1,2), the key Krebs cycle enzymes that generate NADPH reducing equivalents, undergo heterozygous mutations in >70% of low- to mid-grade gliomas and ~20% of acute myeloid leukemias (AMLs) and gain an unusual new activity of reducing the α-ketoglutarate (α-KG) to D-2 hydroxyglutarate (D-2HG) in a NADPH-consuming reaction. The oncometabolite D-2HG, which accumulates >35 mM, is widely accepted to drive a progressive oncogenesis besides exacerbating the already increased oxidative stress in these cancers. More importantly, D-2HG competes with α-KG and inhibits a large number of α-KG-dependent dioxygenases such as TET (Ten-eleven translocation), JmjC domain-containing KDMs (histone lysine demethylases), and the ALKBH DNA repair proteins that ultimately lead to hypermethylation of the CpG islands in the genome. The resulting CpG Island Methylator Phenotype (CIMP) accounts for major gene expression changes including the silencing of the MGMT (*O*^6^-methylguanine DNA methyltransferase) repair protein in gliomas. Glioma patients with IDH1 mutations also show better therapeutic responses and longer survival, the reasons for which are yet unclear. There has been a great surge in drug discovery for curtailing the mutant IDH activities, and arresting tumor proliferation; however, given the unique and chronic metabolic effects of D-2HG, the promise of these compounds for glioma treatment is uncertain. This comprehensive review discusses the biology, current drug design and opportunities for improved therapies through exploitable synthetic lethality pathways, and an intriguing oncometabolite-inspired strategy for primary glioblastoma.

## 1. Oncometabolites: Introduction

To compensate for an increased demand for energy, biosynthetic precursors, and increased macromolecular synthesis, cancer cells enforce a metabolic switching from oxidative phosphorylation to aerobic glycolysis. Many oncogenes such as the *c-myc*, *Ras and PI3K-mTOR*, facilitate a deranged cancer metabolism through a metabolic stress and glutamine addiction [1]. Similarly, the *p53* tumor suppressor increases the expression of *glutaminase 2*, *PUMA*, *TIGAR and glucose transporter 1 and 4 genes* to alter the metabolism [2]. Hallmark cancer mutations have been observed in TCA cycle genes such as the *fumarate hydratase and succinate dehydrogenase*, which are characterized by a loss of function resulting in elevated succinate and fumarate levels in some rare cancers [3]. More recently, mutations in *isocitrate dehydrogenase I and II (IDH1/2)* in cancers have gained wide attention. In contrast to other metabolic enzymes, the IDH1/2 mutants acquire a new function of generating D-2HG. Thus, a new concept that certain metabolites can promote tumorigenesis by either acting as oncogenic signaling molecules, altering the gene expression or modulating the epigenome has emerged and such compounds have received the designation of “oncometabolites”. For example, individuals with germline-acquired mutations in fumarate hydratase develop hereditary leiomyomatosis and renal cell cancer. Fumaric acid and its esters such as the dimethyl fumarate can act as weak electrophiles and conjugate with free or protein-bound cysteines [4]. Such conjugations of fumarate have been shown to underlie increased ferritin synthesis, iron signaling, NRF2 and FoxM1 activations that confer constitutive proliferative signals [5]. Similarly, D-2HG accumulation in IDH-mutant cancers causes changes in oxygen sensing, collagen biosynthesis and epigenetic landscape of the tumor genome. This review summarizes the biochemical and molecular changes associated with IDH mutations, how they contribute to the initiation and progression of cancer, and opportunities for molecular targeting. The various mutant IDH specific inhibitors under development and in clinical trials and unique therapeutic strategies to exploit the beneficial aspects of IDH mutations are also discussed.

## 2. Isocitrate Dehydrogenases (IDHs)

Isocitrate dehydrogenase (IDH), a Krebs cycle enzyme, belongs to a large family of α-hydroxyacid oxidative β-decarboxylases, and it catalyzes the oxidative decarboxylation of isocitrate to alpha-ketoglutarate (α-KG) in a reversible reaction using NAD^+^ or NADP^+^ and Mg^2+^ as cofactors. IDH1 and IDH2 are similar in dimeric structure, function, and evolutionary aspects; however, they differ with the tetrameric IDH3 in structure and functions. IDHs play a crucial role in cellular protection against oxidative stress by generating more than half of reducing equivalent NADPH in cells [4], which is essential for recycling GSH and other redox functions [6]. IDH is also essential in regulating the glutamine and glutamate metabolism [7].

IDH1 is localized predominantly in cytoplasm and peroxisomes and [8]. It serves as one of the major sources of cytosolic NADPH and plays a significant role in lipid and carbohydrate metabolism [9] and thereby protects the cells from bioenergetic and oxidative stresses. It also mediates the de novo lipogenesis through oxidative carboxylation of α-KG to isocitrate under hypoxic conditions [8].

IDH2 is localized in mitochondria [8] and shares almost 97% sequence homology with IDH1. It regulates Krebs cycle and serves as one of the major sources of mitochondrial NADPH. Rest of the functions are similar to IDH1 in protecting cells from various stresses [10].

**Figure 1:**
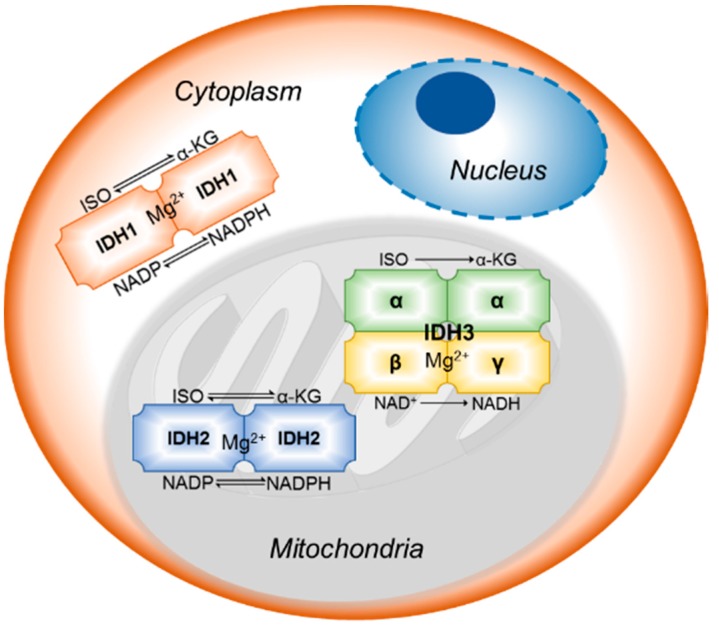
Subcellular location, cofactors, and the reactions catalyzed by IDH isozymes.

IDH3 is an NAD^+^ dependent isozyme located in mitochondria with essential roles in Krebs cycle. IDH3 catalyzes an irreversible reaction unlike its counterparts and is regulated by allosteric effectors [8]. Also, in contrast to IDH1 and IDH2, the heterotetrameric IDH3 is not subjected to cancer mutations (Figure 1).

### 2.1. Mutant IDHs

Cancer whole-genome sequencing efforts from Parson and colleagues revealed the presence of heterozygous, somatic, monoallelic, missense, point mutations in IDH1 in a glioblastoma (GBM) patient in 2008 [11]. Later in 2009, it was found in grade II and III gliomas [12] and for the first time in an acute myeloid leukemia (AML) [13] patient followed by other patients with IDH2 mutations [14]. More recently, IDH1/2 mutations were found in chronic myeloproliferative cancers and myelodysplastic syndromes [14]. The most common mutations include R132H of IDH1 and R172H of IDH2; both these mutations are analogous and change key arginine residues essential for enzyme binding to the substrate isocitrate at the active sites [11]. IDH1 has 3 evolutionally conserved arginine residues, R100, R109, and R132, similarly, IDH2 has R140, R149, and R172. The most common IDH1 mutations include R132H and R132C, and common IDH2 mutations are R172K, R172M, and R140Q. Mutations of IDH1/2 at various loci are summarized in Table 1. IDH1 gene mutations in gliomas exhibit two unique features: the lack of loss of heterozygosity (LOH) and the lack of apparent inactivating mutations such as frameshift or truncations [15].

### 2.2. Mutation Induced-Changes in Enzymatic Activity of IDH

Initially, it was assumed that IDH mutations cause dominant negative inhibition of wildtype IDH activity [22], causing a drop in α-KG levels and consequently cellular signaling pathways that are sensitive to this metabolite are disrupted in oncogenesis. Later, the landmark studies by the Agios Pharmaceuticals, demonstrated that IDH mutations are not just loss of function mutations, but also a gain of new enzymatic activity which leads to the reduction of α-KG to D-2 hydroxyglutarate (D-2HG) [23,24] consuming the NADPH instead of the backward reaction (carboxylation of α-KG back to isocitrate). R132 of IDH1 and R172, R140 of IDH2 form hydrogen bonds with the β-carboxyl moiety of isocitrate substrate and mutations in these residues favor the formation of α-KG → D-2HG instead of isocitrate to α-KG [24]. The mutant IDH cannot catalyze the normal reaction in either direction. Mutations in IDH lead to unusual metabolic patterns and accumulation of oncometabolite, D-2HG [23], which induces biochemical, epigenetic alterations that drive the cancer pathogenesis. Rendina et al. showed that mutant IDH enzymes prefer an ordered sequential mechanism, whereas the wildtype enzymes display a random sequential kinetic mechanism in steady state measurements of the reductive chemical reaction (α-KG → isocitrate vs. α-KG → D-2HG) [25]. The existence of the heterodimeric IDH1 protein (due to the heterozygous type of mutations) suggests the importance of the wildtype subunit in facilitating the mutant reaction. Genetic knock-in experiments introducing an IDH1 R132H encoding mutation into the genome of HCT116 cells demonstrate that the heterozygous IDH1 R132H/*Wt* (wild-type) protein induces genome-wide alterations in DNA methylation. The heterodimeric IDH1 protein may behave differently biochemically as compared with the mutant homodimer. Therefore, the heterozygous R132H mutation is a unique and intriguing target for therapeutic intervention [26]. It has been claimed that *Mt* IDH inhibits *Wt* IDH through heterodimer formation [15]. Ward and colleagues demonstrated that allelic and subcellular compartment differences can regulate the potential for IDH mutations to produce 2HG in cells [27]. Zhang et al. reported that IDH1/2 mutations target a key hallmark of cancer by deregulating cellular metabolism in glioma [28]. These point mutations are reported to restructure many vital residues in the active site of IDH and thus, the affinity for isocitrate and NADP decreases and the affinity for α-KG and NADPH increases yielding a new metabolite D-2HG, which further facilitates oncogenesis, and hence, the D-2HG was described as an “oncometabolite” [29].

### 2.3. Mutant IDH Mediated Oncogenesis

**Figure 2:**
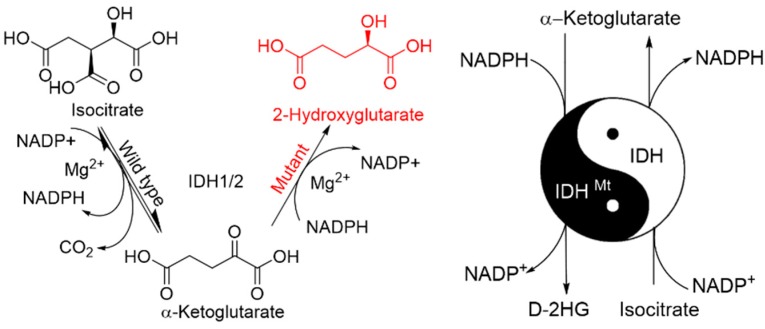
Mutations in the active site of IDH1 and IDH2 lead to a neomorphic enzyme activity. Wildtype IDH catalyzes the conversion isocitrate to α-KG, at the same time reduces NADP^+^ to NADPH and produces CO_2_. R132 of IDH1, R140, and R172 of IDH2 form hydrogen bonds with β-carboxyl of isocitrate substrate. Mutations to these residues cause the enzyme to convert α-KG to D-2HG and NADPH to NADP^+^ instead. The right half of the Figure shows an oncogenic IDH1 dimer made of a wildtype and a mutant subunit that exists in cancer tissues and generates the D-2HG.

Mutation can be the driver of the oncogenesis or an incidental consequence in the tumor progression. IDH mutations are observed as one of the early events in carcinogenesis. It is unclear whether it is mutant IDH or D-2HG or decrease in α-KG or NADPH levels or all of this play role in gliomagenesis. Recent studies, however, indicate that aberrant levels of D-2HG competitively inhibits the α-KG dependent dioxygenases and mediates tumorigenesis. Millimolar amounts of D-2HG is estimated to accumulate in patients with IDH mutations exceeding its elimination mediated by a D-2HG dehydrogenase. D-2HG is identical to α-KG except that the hydroxyl group in D-2HG replaces C2 carbonyl group from α-KG (Figure 2). Thus, D-2HG competitively inhibits various enzymes that are dependent on α-KG and presents itself as an “Achilles heel” in cancer. Oncometabolites such as D-2HG (*Mt* IDH), fumarate (mutant fumarate dehydrogenase) and succinate (mutant succinate dehydrogenase) accumulate at high levels and can target the α-KG dependent dioxygenases [30]. The prevalence and frequency IDH1/2 mutations in CNS, AML, and other cancers are summarized in Table 2.

### 2.4. Is Mutant IDH an Oncogene or a Tumor Suppressor Gene?

It is unclear whether mutant IDH functions as an oncogene or tumor suppressor gene. There is data supporting both these possibilities, but more so for an oncogenic function. Functional studies of IDH1 and IDH2 mutations revealed that these mutations reduce the ability of IDH1 and IDH2 to convert isocitrate to α-KG [12], which led to the speculation that IDH1 and IDH2 are tumor suppressor genes (TSG) with a propensity to develop dominant negative point mutations. Loss of heterozygosity (LOH) at the IDH1 locus in gliomas [15,16] and leukemias [33], as well as monoallelic expression of IDH1 in gliomas, are well known. In addition, a recent report claims mutations in IDH results in loss of function without elevation of D-2HG [34] supporting that at least in some cases IDH1/2 may function as a typical tumor suppressor gene [35].

Nevertheless, the facts that IDH harbors only point mutations, but no frame-shifts, deletions or non-sense mutations indicate an oncogenic gain of function. Further, siRNA knockdown studies of IDH1 and IDH2 resulted in slow a growth of cancer cells. Promoter methylations of IDH1 and 2 are also non-existent [35]. In addition, it is clear that IDH1 mutations are monoallelic, confined to a single residue in the enzyme’s active site, unlike any other typical tumor suppressor gene. Transfection of mutated IDH into normal cells increased the proliferation, colony formation indicating the aberrant IDH1 to be a driver of the gliomagenesis [36,37,38]. Efforts of Sasaki et al. characterized the phenotype of a conditional knock-in mouse model in which they inserted IDH1 R132H into endogenous IDH1 locus and expressed it either in the brain or hematopoietic system of mice. Brain-specific expression resulted in perinatal lethality of mice due to cerebral hemorrhage with no indication of tumors but if mice had lived longer, they would have developed tumors. In contrast, the hematopoietic system specific IDH1 R132H knock-in mice had a normal lifespan with no malignancy. In brain cells isolated from these mutants, D-2HG produced by the activity of the abnormal IDH enzyme functioned as an oncometabolite that induced HIF target gene transactivation, disrupted collagen maturation, and impaired the basement membrane structure [39].

### 2.5. Mutant IDH-Derived D-2HG Is an Oncometabolite

D-2HG, D-2 hydroxyglutarate is the common enantiomer of 2-hydroxyglutarate and is formed by various metabolic enzymes; however, its levels are kept low by the housekeeping enzyme D-2HG dehydrogenase (D-2HGDH) that recycles it back to α-KG. Elevated levels of D or L-2HG levels also occur in a rare, inherited neurological disorder called HG aciduria, in patients with homozygous, inactivating mutations in the gene coding for D-2HGDH or L-2HGDH. Patients with D-2HG aciduria do not have a high risk of cancer; however, it is important to note that these patients die in their infancy, therefore, the cancer risk cannot be assessed accurately [40].

IDH mutant cancers exclusively produce D-2HG at high concentrations up to 30–50 mM compared to the <0.1 mM in normal cells; these numbers also translate to 54.4 mg vs. 0.1 mg of D-2HG/gram protein [23,41,42,43,44]. The Kcat of *Mt* IDH is 1 × 10^3^ s^−1^ [20] whereas the recombinant D-2HGDH has an estimated Kcat of 0.8 s^−1^ [45] which explains the obvious reason for accumulation of D-2HG. Losman et al. reported that D-2HG is sufficient to promote leukemogenesis in a reversible manner and they observed that exogenous D-2HG recapitulates the effects that are seen with IDH1 R132H mutations [46].

### 2.6. Incidence of IDH1/2 Mutations

IDH1 mutations occur somatically in >70% in grade II–III gliomas and secondary GBMs, and 8.5% of AML cases [24]. These mutations were also reported later in cancers of the colon, prostate [47]. IDH mutations are characterized by some distinct clinical features. First, it is an early if not an initial event in gliomagenesis [48] and has significant implications for progression, malignancy, and response to standard chemotherapy. Second, their occurrence is restricted to a spectrum of tumors such as grade 2/3 gliomas, secondary GBM but it is rare in primary GBM [16]. Third, the presence or absence of IDH mutations in GBM, AML and cholangiocarcinoma patients has generated much interest in elucidating their significance to oncogenic progression and clinical outcome [49]. Grade II/III gliomas without mutant IDH are considered “pre-GBMs” for their poor prognosis compared to mutant IDH tumors of the same grade [50]. Fourth, IDH mutations prevail in younger brain tumor patients [51], most commonly ages between 20 and 40, with surprising preference to the frontal lobe tumors and associated with less contrast enhancement and less necrosis compared to the non-IDH mutant gliomas [52]. IDH1/2 mutations are mutually exclusive in nature [53]. In general, p53 mutations are more frequent in low-grade gliomas such as astrocytoma and anaplastic astrocytomas and 1p19q codeletions are more seen in oligodendroglioma and anaplastic oligodendroglioma. Interestingly, IDH1/2 mutations are found at relatively similar frequency [12] as seen in p53 mutations and 1p19q codeletions suggesting the contribution of IDH mutations towards the pathogenesis of both astrocytic and oligodendroglial tumors [54]. Ahmad et al. and Im et al. reported the association of DNMT3A and IDH1/2 mutations in AML [55,56]. Mutations of epidermal growth factor receptors (EGFR) and deletion of PTEN are more common in high-grade primary GBMs and are found to be mutually exclusive [57]. Glioma-CpG Island methylator phenotype (G-CIMP) tumors belong to a proneural subgroup, are more prevalent in low-grade glioma, display distinct copy number alterations and are tightly associated with IDH1 somatic mutations [58]. Hypermethylation of CpG dinucleotide sequences, which are seen in the promoter sequences of tumor suppressor genes, and repair genes such as *O*^6^-methylguanine methyltransferase (MGMT), seems to be closely associated with IDH mutations [59,60]. Hypermethylation of DNA may be a direct consequence of epigenetic modification driven by IDH mutations as discussed in subsequent sections.

The World Health Organization (WHO) in 2016 for the first time considered molecular parameters in addition to the histology for the CNS tumor classification and incorporated new entities that are defined both by the histology and molecular features, including GBM, IDH-wildtype, GBM, IDH-mutant along with other classes [3]. Various classes of CNS tumors such as diffuse astrocytoma, anaplastic astrocytoma, glioblastoma, oligodendroglioma and anaplastic oligodendroglioma were further subclassified into IDH wildtype or mutant classes along with other molecular features.

## 3. Clinical Relevance

IDH mutation status or D-2HG levels are employed in the clinic as a diagnostic tool and has been claimed multiple times for its predictive and prognostic value as well. Clinical studies involving chemotherapy for recurrent malignant oligodendroglioma carried out in North America (RTOG 9402) and Europe (EORTC 26951) clearly indicated higher overall survival benefits (9.4 years vs. 5.7 years) for both anaplastic oligodendroglioma and oligoastrocytoma patients who in addition to radiation therapy received chemotherapy of procarbazine, CCNU/lomustine, and vincristine. Among the three agents of above regimen, CCNU and procarbazine belong to the class of alkylating agents.

### 3.1. Diagnostic Marker

IDH mutant status determination has emerged as a standard clinical practice in gliomas. Direct sequencing-based technologies and immunohistochemistry (IHC) are the few methods that are employed currently to diagnose the IDH mutations in gliomas. Genotyping, including allele-specific PCR, are also performed on the DNA isolated from the brain samples. With the emergence of sensitive and mutant-specific IDH1 R132H antibodies [17], IHC has evolved as a better, accessible, and economical option compared to the direct sequencing. IHC is also used to confirm the completeness of the surgical resection and for the analysis of post-therapy biopsy samples. IHC also allows differential diagnosis between gliomas and non-neoplastic CNS lesions, between gliomas and non-glial CNS tumors and within glioma subtypes. Catteau et al. developed and validated a new real-time quantitative polymerase chain reaction (PCR) assay for single-step detection of IDH1 R132H and other mutations in formalin-fixed paraffin-embedded (FFPE) glioma samples [61].

Measurement of serum or urine D-2HG levels in AML patients and its decreased levels after treatment has been validated as a diagnostic marker. Serum D-2HG levels of ≥170 ng/mL could predict the presence of an IDH/2 mutation with an 83% sensitivity and 90% specificity [62]. However, serum levels in glioma patients do not seem to correlate with tumor size or tumor grade or mutation status [63,64]. Direct LC/MS or GC/MS analysis has also been used to quantitate the levels of D-2HG [65]. Blood samples contain measurable D-2HG, which helps as a prognostic marker in AML [66]. A novel technique, which combines the co-amplification at a lower denaturation temperature and digital polymer chain reaction was reported to detect IDH R132H in glioma patients [67]. Balss et al. developed an inexpensive and sensitive enzymatic assay for quantitative analysis of D-2HG which can be employed for the cell, tumor lysates or paraffin-embedded tumors, and it is based on the conversion of D-2HG to α-KG in the presence of enzyme D-2HGDH and NAD^+^. D-2HG levels are directly proportional to the fluorescence intensity of resorcinol formed from the resazurin in a reaction mediated by the NADH formed in situ in the earlier step [68].

Noninvasive techniques such as magnetic resonance spectroscopy (MRS) imaging of the brain to measure the D-2HG levels are also under consideration as it can enable surgeons to predict the pre-operative tumor histology, interoperative imaging to better visualize the tumor margins, postoperative analysis of radio/chemotherapy and early detection of disease recurrence. Choi et al. reported the noninvasive detection of D-2HG by proton MRS [42]. Nagashima et al. recently extended MRS detection to glutamate along with D-2HG. Metabolic profiling revealed glutamate levels were significantly decreased in IDH mutant cells compared to the non-IDH mutant counterparts. A stereotactic navigation-guided operation was performed to resect tumors tissues and achieved 72% sensitivity with 96% specificity in 47 patients with gliomas [69].

### 3.2. Predictive Marker

IDH mutations have advanced as a predictive marker as well, especially in glioma therapy. IDH mutant patients respond better to temozolomide therapy compared to patients with non-IDH mutant gliomas. Surprisingly, IDH mutant status does not predict response to procarbazine, lomustine, and vincristine in patients with anaplastic oligodendroglioma providing insights into the possible inactivation of MGMT because of CIMP induced by IDH mutations. With the emerging treatment modalities and sophisticated surgical techniques that are available in the clinic, the predictive value of IDH mutations enables the neuro-oncologists to tailor patient-specific drug regimens.

### 3.3. Prognostic Marker

IDH mutations serve an important role as prognostic markers in the clinic. IDH mutations are the most powerful single prognostic factor for improved overall survival (OS, 3-fold), followed by age, tumor type, and MGMT promoter methylation status. IDH mutations have been reported to confer an independent favorable prognosis in WHO grade III oligodendroglioma and a 1p/19q co-deletion and MGMT promoter methylation were strongly associated with IDH mutations [70]. Post-treatment serum levels of D-2HG levels <200 ng/mL correlates with longer survival in IDH mutant AML patients and is also used as a prognostic marker [71]. The association of IDH mutations with a better patient overall survival (OS) and progression-free survival (PFS) has been reported in many studies. Existing clinical statistics claim that a median overall survival of the is 31 months for the secondary GBM patients with IDH mutations compared to 15 months for those without mutations, whereas patients with IDH mutant anaplastic astrocytoma have 65 months of median overall survival compared to 20 months in the counterparts [12]. The events of longer survival with IDH mutations are commonly seen in lower grade gliomas such as oligodendroglioma (94%) and less so in astrocytoma (72%) or mixed tumors (83%) [72,73,74,75,76,77] and is reported as a weak predictor of a long time overall survival in GBMs [78]. In the patients with intrahepatic cholangiocarcinoma, harboring IDH mutations, it took longer time for tumor recurrence after surgical resection and helped for longer overall survival compared to those without IDH mutations [79]. In AML, IDH2 but not IDH1 is associated with longer survival [80]. Various postulations are reported explaining this survival benefit and one such hypothesis is silencing of MGMT gene induced by the “glioma-CpG island methylator phenotype [81] (G-CIMP)” induced by mutant IDH [82,83,84,85,86] though another report contradicted the same [69]. Studies of Ohba and colleagues revealed that mutant IDH1 increases Rad51 mediated homologous recombination and further leads to temozolomide resistance in cancer cell lines [87].

## 4. Biochemical Alterations Specifically Occurring in Tumor Tissues Due to IDH Mutations

IDH mutations appear to promote a permissive state for cell transformation. They result in a profound increase of cellular methylome associated with epigenetic changes which are tightly linked to the oncogenesis. These mutations are also reported to block the cell differentiation and facilitate increased cell proliferation [37]. Metabolic acidosis is considered to be the common feature of the tumor microenvironment. Basing on this notion, Nadtochiy et al. hypothesized that pH regulates the 2-HG formation and the results strengthened their hypothesis at the cellular, mitochondrial and isolated enzyme levels [88]. IDH mutations are tied to changes in collagen maturation, hypoxia-inducible factor (HIFα) and DNA damage response, some of which have been discussed in sections below.

### 4.1. D-2HG Inhibits α-KG Dependent Dioxygenases

**Figure 3:**
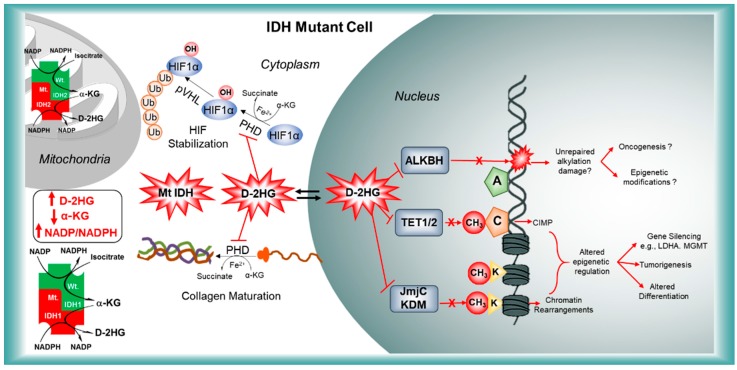
A wide variety of molecular changes that occur due to the competitive inhibition of α-KG-dependent dioxygenases by D-2HG, namely the prolyl hydroxylase domain 1 (PHD) resulting in defective collagen maturation and stabilization of hypoxia-inducible factor (HIF1), inactivation of ALKBH repair that promotes tumorigenesis, inhibition of TET1/2-mediated catabolism of 5′-methylcytosine and Jumanji-domain containing histone demethylases (JmjC KDM) that enforce epigenetic modifications of DNA and consequently the gene silencing in IDH1/2 mutant cells are shown.

α-KG Dependent dioxygenases are the oxidoreductases that catalyze hydroxylation, using molecular oxygen to add both atoms of O_2_ to the substrate and utilize α-KG, a co-substrate and ascorbate and iron as cofactors yielding an oxidized product along with succinic acid and CO_2_ as byproducts. There are nearly 80 various α-KG dependent dioxygenases in human tissues and they sit at the intersection of nutrient availability and metabolism where they have the potential to regulate gene expression and growth in response to the substrate and co-factor abundance. They regulate the fundamental cellular processes by catalyzing the hydroxylation or demethylation of DNA, RNA or protein components [27]. These enzymes are JmjC domain-bearing histone demethylases (KDMs), prolyl hydroxylase (PHD) which regulates hypoxia-inducible factor (HIF), DNA demethylases such as the 5-methylcytosine hydroxylases (TET family), prolyl/lysyl hydroxylase (PHDs/LHDs), ALKBH4 etc. which are involved in histone modifications, stimulating angiogenesis, aberrant DNA methylation, abnormal collagen maturation and repair of oxidative DNA damage respectively. Thus, it is believed that D-2HG mediated inhibition of α-KG dependent dioxygenases is one major reason through which IDH1/2 mutations contributing to the pathogenesis of either glioma or leukemia. Figure 3 summarizes how D-2HG inhibits α-KG dependent dioxygenases.

#### 4.1.1. IDH Mutations-Epigenetic Alterations

Gene expression is regulated at various levels and one such is epigenetic level includes the post-translational modifications of histones and DNA. The examples are methylation/acetylation/phosphorylation of lysines in histones, methylation/acetylation of cytosines in DNA. Disruption of normal patterns of epigenetic markers is highly associated with tumor progression. 5-methylcytosine hydroxylase (TET) and JmJC domain-containing histone lysine demethylase (KDMs: JMJD2A/2C etc.) are few such enzymes that utilize α-KG as a co-substrate and play key roles in generating and maintaining epigenetic landscapes and thus, are the targeted by D-2HG in IDH mutant cells.

- DNA Methylation

DNA methyltransferases (DNMTs) are the enzymes that transfer methyl to the 5th carbon of cytosine using *S*-adenyl methionine as the methyl donor [89]. D-2HG competitively inhibits TET family enzymes [90] and promotes hypermethylation of DNA [91]. TET family of dioxygenases catalyze the hydroxylation of 5-methylcytosines (5-mCs) in DNA to form 5-hydroxymethylcytosine (5-hmC) and further 5-formylcytosine (5-fC) and 5-carboxycytosine (5-caC) finally leading to the demethylation of cytosine and restoration of the base in its original form [92]. Though the mechanisms that regulate the activity of TET are not well known, it is clear that TET plays a major role in maintaining the DNA methylation patterns [93]. TET itself controls cell differentiation and embryonic stem cell function by regulating the expression of genes that maintain pluripotency [94]. IDH mutation derived D-2HG interferes with TET driven DNA demethylation, causing elevated 5-methylcytosine levels, resulting in the hypermethylated signature on DNA, a characteristic feature of less differentiated cells [95]. Xu et al., also demonstrated that ectopic expression of IDH1/2 mutations reduces 5-hmC levels [90].

In gliomas, a tumor phenotype known as the CpG island methylator phenotype (CIMP) is highly associated with IDH1 mutations and Turcan et al. reported that IDH1 mutation is sufficient to establish the CIMP [96]. It is a powerful determinant of glioma pathogenicity and patients with this phenotype are mostly younger and had better survival, features that were also seen in IDH mutant cases. The introduction of IDH1 R132H into astrocytes through lentiviral vectors also demonstrated DNA hypermethylation, conversely, the opposite was seen at specific loci when *Wt* IDH1 was overexpressed [96]. Hypermethylation of DNA, especially in the promoter sequence of mRNA leads to repression of gene transcription as methylation of CpG islands, altering the affinity of transcription factors with promoter sequences. Methylated DNA binds to a protein complex which comprises of a methyl binding protein (MBP), a corepressor molecule (CR) and a histone deacetylase (HDAC). When this complex binds to methylated DNA, the histones become deacetylated through the HDACs, resulting in chromatin compaction making the DNA inaccessible to the transcription machinery. Hypermethylation of CpG islands are predominantly seen in promoter sequences of tumor suppressor genes, DNA repair genes (hMLH1, MGMT), cell cycle genes (p16INK4a, p15INK4b, p14ARF), apoptosis (DAPK), cell adherence (CDH1, CDH13), detoxification (GSTP1) [97]. It was thought that inhibition of TET2 by mutant IDH is the driver of AML carcinogenesis, but Inoue et al. found significant clinical differences between IDH1 and TET mutant diseases, giving hints of additional mediators in mutant IDH1-induced carcinogenesis [98]. They discovered that mutant IDH1 downregulates the DNA damage sensor, ATM, by altering the histone methylation, which further leads to impaired DNA repair, increased sensitivity to DNA damage and reduced hematopoietic stem cell self-renewal, independent of TET2.

- Histone Methylation, Cell Differentiation

**Figure 4:**
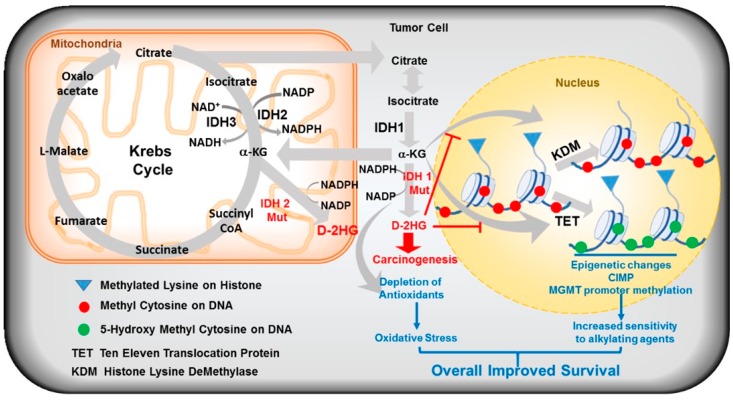
Major consequences of IDH 1 mutations in lower grade gliomas. We propose that an enhanced oxidative stress due NADPH consumption for D-2HG production by mutant IDH1 and the generation of CpG island methylator phenotype (CIMP) due to a competitive inhibition of TET1/2 enzymes and histone demethylases (KDM) explain the superior therapeutic responses seen in these patients. We also postulate that promoter methylation silencing the MGMT DNA repair gene contributes an increased sensitivity to temozolomide and other alkylating agents used in treatment. MGMT removes the cytotoxic lesions and is a major resistance determinant in gliomas. Accumulation of methylcytosine and 5-hydroxymethylcytosine (due to TET inhibition) and histone methylations in the glioma genome are also represented.

Post-translational modifications of histones are important for chromatin reorganization and gene transcription. Various subtypes of histone demethylases are reported to be overexpressed in cancers and were suggested as possible targets for cancer therapy. Chowdhury et al. performed different biochemical, structural, and cellular studies and observed that D/L-2HG inhibit human α-KG dependent dioxygenases such as FIH, PHD2, JMJD2A/C, BBOX, ABH2 but with different potencies ranging from 25 μM to >10 mM [99]. D-2HG inhibits histone demethylases at 24 μM and PHD2 at 7 mM indicating that IDH R132H induced oncogenic pathways may include chromatin modifications. A member of JmJC family proteins has been crystallized in complex with D-2HG and it was found to occupy the α-KG binding pocket [99]. Lu et al. confirmed this observation and further reported that impaired histone demethylation results in a block to cell differentiation. Rosaik et al. found that IDH1 R132H increases apoptotic susceptibility of neuronal stem cells and their successors yielding differentiation deficient mutant cells [100]. Figure 4 summarizes the major changes as we hypothesize to occur in glioma cells due to IDH-1 mutations.

#### 4.1.2. HIF Signaling and Metabolism

Hypoxia Inducible Factor (HIF) is a transcription factor that, when overexpressed, is associated with malignant progression and poor outcome in several cancers [101]. HIF is composed of an oxygen sensitive α-subunit (HIF 1α) and a constitutively expressed β-subunit (HIF 1β) [102]. HIF has a wide range of target genes that promote cell adaptation to low-oxygen tension (hypoxia). HIF regulates various genes that modulate angiogenesis, glycolysis, growth factor signaling, apoptosis and metastasis [103]. During normal oxygen levels, HIF 1α is destabilized by post-translational hydroxylation of two prolines by a prolyl hydroxylase (HIF-PHD or EGLNs), which is an α-KG dependent dioxygenase. Prolyl hydroxylation leads to pVHL (von-Hippel-Lindau protein)-dependent ubiquitination and proteasomal degradation of HIF 1α [104]. Xu et al. and Hewitson et al. demonstrated that D-2HG inhibits the HIF-PHDs and thereby stabilize the HIF 1α [90,105]. HIF 1α was shown to be upregulated in IDH mutant cells or cells treated with exogenous D-2HG or in Nestin-Cre mice bearing brain tumors with IDH R132H mutations [12,36,91]. However, it is pertinent that D-2HG is a relatively a weak inhibitor of HIF-PHD compared to JMJD2A. In addition, Metellus et al. and Williams et al. found that there was no correlation between IDH mutations and HIF 1α stabilization in patients with IDH mutant gliomas [101,106]. In some human astrocytes, colorectal or leukemic cells, the expression of IDH R132H has been shown to attenuate the HIF levels [107]. Many target genes of HIF are expressed at lower levels in IDH mutant gliomas [108,109]. Chesnelong and colleagues reported that HIF 1α responsive, glycolysis-related genes such as SLC2A1, PDK1, LDHA and SLC16A3 are under-expressed in IDH mutant gliomas. LDHA is essential for glycolysis and is overexpressed in cancers. IDH1/2 mutation-driven downregulation of LDHA prevents glycolytic switch (Warburg effect) and limits the growth of gliomas. Silencing of LDHA was associated with hypermethylation of its promoter site [108]. Collectively, these findings highlight the metabolic cross-talk between IDH mutations and hypoxic adaptation.

#### 4.1.3. IDH Mutations-Collagen Maturation

Sasaki et al. recently tried to establish the physiological significance of IDH mutations in vivo by generation and characterization of brain-specific IDH1 R132H conditional knock-in mice and found that IDH1 mutations result in hemorrhage and perinatal lethality [39]. They also observed decreased ROS levels despite increased NADP^+^/NADPH ratios. D-2HG accumulation lead to inhibition of prolyl/lysyl hydroxylation (PHD(C-P4H1-3)/LHD(PLOD-3)) of collagen, resulting in defective/immature collagen fibers, which could result in endoplasmic reticulum (ER) stress and explain the embryonic lethality. Impaired collagen maturation also led to fragile basement membranes which could potentially favor tumor invasion and metastasis. Further, increased levels of immature collagen type IV were found in IDH mutant cells such as HT1080 compared to the non-mutant cells [110]. Overexpression of D-2HGDH in HT1080 cells significantly decreased the levels of soluble collagen type IV but not in non-mutant cells. Inhibition of collagen maturation also resulted in an altered tumor microenvironment and defective angiogenesis. Another study showed that D-2HG either produced from mutant IDH or through glutamine anaplerosis resulted in increased trimethylation of histone H3 lysine 4 in the promoter region of ZEB1, a master regulator epithelial-mesenchymal transition [111].

#### 4.1.4. IDH Mutations-ALKBH DNA Repair Enzyme

ALKBH (Alkylation repair homologs) family proteins are homologs of AlkB, an *E. coli* enzyme that repairs alkylated DNA and RNA. These are Fe(II) and α-KG dependent dioxygenases that remove the alkyl and exocyclic bridged adducts from nucleic acid bases by oxidative dealkylation [112]. Typically, the bases are returned to their unmodified forms at the end of the reaction. Among the nine human AlkB homologs, ALKBH2 and ALKBH3 are the only enzymes that repair DNA or RNA. Recently, D-2HG was shown to inhibit ALKBH2 in a manner similar to the TET enzymes [113]. Cells expressing mutant IDH displayed reduced repair kinetics, accumulated more DNA damage, and were sensitized to methyl methanesulfonate (MMS), MNNG and busulfan suggesting that IDH1/2 mutations may increase the therapy-induced alkylation damage. However, whether the base modifications induced by therapeutic alkylating agents are substrates for the ALKBH2 and 3 is not known. Any such unrepaired DNA damage may contribute to carcinogenesis in IDH1/2—mutant cells.

#### 4.1.5. Mutant IDH-Induced Oxidative Stress

IDH1 and IDH2 play a cytoprotective role against ROS, radiation, and other damage lesions [114]. As such, protection against the cellular stress induced by selenium or staurosporine has been reported. The reducing equivalent, NADPH, generated as a byproduct is important in this cytoprotection. NADPH is a vital component in maintenance redox homeostasis, biosynthesis of lipids, nucleotides, and recycling of oxidized glutathione to reduced GSH. α-KG, an antioxidant by itself along with the GSH and thioredoxin antioxidant systems remain downregulated in IDH-mutant cells. The attenuation of NRF2-mediated gene expression also contributes to the increased redox imbalance found in cancers.

## 5. Therapeutic Avenues

### 5.1. Selective Mutant IDH Inhibitors

This class of drugs is based on the principle that mutant IDH functions as an oncogene. It has proved to be a compelling drug target for new therapies for glioma and AML patients. Contrary to the typical oncogenes, the active sites of this metabolic enzyme are amenable to small molecule targeting [115]. The crystal structures of IDH1 R132H containing isocitrate have provided some understanding of the change of mutant catalytic function [8]. Some of the reported inhibitors have crystallographic data and can be broadly classified into two classes, those that occupy the active site of the protein and those that occupy a remote allosteric site which renders the protein inactive through a conformational change.

Mutant IDH selective inhibitors suppress the D-2HG production and have been shown in multiple studies to reverse the epigenetic changes and cellular differentiation by inducing cell-cycle arrest and thereby keeping the tumor progression in check. There has been a huge interest and thrust in the development of small molecule inhibitors and allosteric modulators against the mutant IDH enzymes, with the hope of attenuating the oncometabolite levels and arresting the rate of tumorigenesis and ultimately a therapeutic cure. A complete list of drugs synthesized, those under development and limited trials, along with their chemical structures and sources of manufacture and citations are shown in Table 3. Many of these drug-discovery efforts are summarized below.

Popovivi-Muller et al. identified IDH1 mutant-specific inhibitors through a high-throughput screening (HTS) against IDH1 R132H mutant protein homodimer and found phenyl-glycine scaffold containing compound **1** binding in a reversible and competitive manner with R132H with an IC_50_ of 0.09 μM. Further development of compound **1** yielded AGI-5198 (Compound **2**), the first reported mutant IDH1 inhibitor that showed robust in vivo reduction of D-2HG levels in U87 R132H and HT1080 R132C cell lines and in U87 R132H tumor xenograft model [119]. Wang et al. from Agios Pharmaceuticals, developed a small molecule, AGI-6780 (Compound **4**), that potently and selectively inhibited the tumor-associated mutant IDH2 R140Q (IC_50_ = 170 nM) [116]. Crystal structure of AGI-6780 complexed with IDH2 R140Q revealed that inhibitor binds allosterically at the dimer interface; in addition, AGI-6780 induced differentiation of leukemia cells in vitro by reversing the mutation induced histone and DNA hypermethylation [121]. Rohle et al. identified a selective IDH1 R132H inhibitor, AGI-5198 (K_i_ = 0.07 and 0.16 μM vs. R132H and R132C, K_i_ > 100 μM vs. *Wt* IDH1), through HTS and found it blocked the production of D-2HG, delayed growth and promoted differentiation by inducing the demethylation of histone H3K9me3 in a dose-dependent manner in TS603 IDH1 R132H cells. Oral administration of AGI-5198 in mice with xenograft subcutaneous tumors significantly reduced intratumoral D-2HG levels. Further immunohistochemical staining of histone methylation and increased expression of astroglial differentiation genes explained the mechanism behind the observed decline in tumor volume [120,138]. Structure refining of AGI-5198 has led to the development of AG-120 (Compound **5**) and AG-221 (Compound **1**) (Agios Pharmaceuticals), orally administrable drugs targeting IDH1 and IDH2 mutations, respectively.

A quantitative high-throughput screening of the R132H mutant IDH1 enzyme and the subsequent optimization of the small molecule hit resulted in probe, ML309 (Compound **7**), which is capable of potent and selective inhibition of mutant IDH1 (K_i_ 96 nM vs. 35 μM for *Wt* IDH) and effectively lowers cell-based production of D-2HG in a U87MG mutant glioblastoma cell line and later it was also shown to inhibit IDH1 R132C with similar selectivity [123]. It had moderate plasma half-life but its impenetrability to BBB probably hindered its further development [139].

Several 1-hydroxy pyridine-2-one compounds (Compound **13**) were identified as inhibitors of IDH1 R132H. A total of 61 derivatives were synthesized, and their structure-activity relationships were investigated [127]. Potent IDH1 R132H inhibitors were identified with K_i_ values as low as 140 nM, while they possess weak or no activity against *Wt* IDH1. Activities of selected compounds against IDH1 R132C were found to correlate with their inhibitory activities against IDH1 R132H, as well as the cellular production of D-2HG. Several inhibitors were found to be permeable to the blood-brain barrier in a cell-based model assay and exhibit potent and selective activity (IC_50_ = 0.26–1.8 μM) against IDH mutant glioma cells. Same research group later identified two 1-hydroxy pyridine-2-one compounds that are potent inhibitors of IDH1 R132H and R132C mutants with K_i_ values as low as 120 nM. These compounds (Compounds **14** and **15**) exhibited >60-fold selectivity against wildtype IDH1 and can inhibit the production of D-2-hydroxyglutaric acid in IDH1 mutated cells [128].

Zou et al. focused on the allosteric site of mutant IDH1 and by utilizing the docking-based virtual screening with docking software and subsequent biological experiments. Out of 200,000 compounds screened, they identified four compounds and among which, FX-03 (Compound **12**) found a selective mutant IDH inhibitor. Although these were not as potent as the earlier compounds, the study paved the way for discovery and development of novel selective mutant IDH1 inhibitors at the allosteric site [126].

A high-throughput screening for selective inhibitors of IDH1 bearing the oncogenic mutation R132H identified VVS (Compound **9**), a bis-imidazole phenol, the first co-crystal structure of mutant IDH1 inhibitor complex (PDB code 4UMX) that reversibly inhibits D-2HG production in cells in a time-dependent manner. Steady-state kinetics and biophysical studies showed that both compounds selectively inhibited mutant IDH1 by binding to an allosteric site and that inhibition was competitive with respect to Mg^2+^. A crystal structure of VVS complexed with R132H IDH1 indicated that the inhibitor binds at the dimer interface and makes direct contact with an Asp279 residue involved in binding of the catalytically essential divalent cation [124]. The inhibitory mechanism suggested that the disorganization of the active site induced by oncogenic IDH mutations not only favors their unusual neomorphic activity but also imposes a neomorphic vulnerability to pharmacological inhibitors directed toward the metal-binding regulatory segment. Structural plasticity caused by the mutations may allow this segment to adopt new, catalytically inactive conformations that are stabilized by drug-like small molecules in competition with the catalytically active conformation stabilized by Mg^2+^.

Crystallographic and biochemical results demonstrated tetrahydro-pyrazolopyridine (THPP) series of inhibitors that can bind to an allosteric site and lock the enzyme in a catalytically inactive conformation, thereby enabling inhibition of different clinically relevant IDH1 mutants. Additional led to the identification of GSK321 (Compound **10**), a potent inhibitor of mutant IDH1 enzymes, with K_i_ values of 4.6, 3.8 and 2.9 nM against R132H, R132C, and R132G respectively with no activity against mutant IDH2 and 46 nM with *Wt* IDH. GSK321 and GSK990 (Compound **11**) displayed a non-competitive mode of inhibition against NADPH and a competitive mode of inhibition with α-KG. Treatment of IDH1 mutant primary AML cells uniformly led to a decrease in intracellular 2-HG, abrogation of the myeloid differentiation, and more immature stem-like cells [125]. Molecularly, treatment with the inhibitors led to a reversal of the DNA cytosine hypermethylation patterns caused by mutant IDH1 in the cells of AML patients.

Wu and colleagues reported on the synthesis, structure-activity relationship, enzyme kinetics, and binding thermodynamics of 2-thiohydantoin compounds (Compounds **17**–**19**) (K_i_ = 0.42 μM) with K_i_ *Wt*/R132H selectivity index of more than 20. These compounds were shown to decrease cellular D-2HG levels, reduce histone methylation, suppress proliferation of stem-like cancer cells in BT142 glioma with IDH1 R132H mutation [130].

An optimized high-throughput assay quantifying consumption of NADPH by IDH1 R132H was implemented to screen 3 million compounds and hits were characterized by Rapid Fire–Mass Spectrometry measuring D-2HG directly. Multiple distinct chemotypes were identified with nanomolar potencies (6–300 nM) in this study. All inhibitors were found to be competitive against α-KG, not NADPH and were inactive against the wildtype IDH1 homodimers. Interestingly, one of the inhibitors, EXEL-9324 (Compound **8**), was found to inhibit the enzymatic activity of IDH1 heterodimer (*Wt*/*Mt*) but not the *Wt*/*Wt* homodimer [140].

A collaborative high-throughput screening of 1.35 million compounds against mutant (R132H) isocitrate dehydrogenase IDH1 led to the identification of a novel series of inhibitors. Elucidation of the bound ligand crystal structure showed that the inhibitors exhibited a novel binding mode in a previously identified allosteric site of IDH1 (R132H). This information guided the optimization of the series yielding submicromolar enzyme inhibitors with promising cellular activity. One compound (Compound **16**) from this series was found to induce myeloid differentiation in primary human IDH1 R132H AML cells in vitro [129].

Recently, another research group from Novartis identified 4-isopropyl-3(2-((1-phenylmethyl) amino)-pyrimidine-4-yl) oxazolidine-2-one as a potent inhibitor of IDH1 R132H after HTS and subsequent hit validation. Synthesis of the four separate stereoisomers identified the (*S*,*S*)-diastereomer, IDH125 (Compound **21**), as the most potent isomer. Potency improvement and modulation of the physicochemical properties identified (*S*,*S*)-oxazolidinone IDH889 (Compound **20**) with good exposure and 2-HG inhibitory activity in a mutant IDH1 xenograft mouse model [131].

A research group from Novartis explored 3-pyrimidin-4-yl-oxazolidin-2-ones (Compound **23**) [133] as mutant IDH1 inhibitors for in vivo modulation of 2-HG production and potential brain penetration. Further, optimization efforts toward the identification of clinical candidate, they found, a potent and selective mutant IDH1 inhibitor IDH305 (Compound **22**). Preclinical characterization of this compound exhibited in vivo correlation of 2-HG reduction and efficacy in a patient-derived IDH1 mutant xenograft tumor model [132].

Heuser et al. from Bayer have developed a novel, highly active, a pan-IDH1 inhibitor with favorable oral pharmacokinetic properties and reasonably brain-penetrant, BAY1436032 (Compound **24**) for clinical evaluation. Its inhibitory potency was evaluated in primary human AML cells and patient-derived AML xenograft models. D-2HG production by mutant IDH1 was effectively inhibited in patient-derived AML cells with all reported IDH1R132 mutations ex vivo by BAY-1436032 with an IC50 between 3 and 16 nM [134,135].

In order to, discover mutant allele-selective IDH1 inhibitors with chemical features distinct from existing probes. A potent (K_i_ = 50 nM) series of IDH1-R132H inhibitors having 8-membered ring sulfonamides as exemplified by the compound BRD2879 (Compound **25**) were identified. BRD2879 inhibited (*R*)-2-hydroxyglutarate production in cells without apparent toxicity. However, the solubility and pharmacokinetic properties of the specific inhibitor BRD2879 prevent its use in vivo, the scaffold presents a validated starting point for the synthesis of future IDH1-R132H inhibitors [141].

Another IDH1R132H inhibitor, clomifene citrate (Compound **27**), was very recently found by virtual screening method, which can selectively suppress mutant enzyme activities in vitro and in vivo in a dose-dependent manner. The molecular docking indicated that clomifene occupied the allosteric site of the mutant IDH1. Enzymatic kinetics also demonstrated that clomifene inhibited a mutant enzyme in a non-competitive manner. Moreover, knockdown of mutant IDH1 in HT1080 cells decreased the sensitivity to clomifene. In vivo studies indicated that clomifene significantly suppressed the tumor growth of HT1080-bearing CB-17/Icr-SCID mice with oral administration of 100 mg/kg and 50 mg/kg per day [142].

The Agios Pharmaceuticals in association with Celgene has made much progress and their IDHIFA^®^ (AG-221, Enasidenib, compound **1**) is the first FDA approved mutant IDH2 selective inhibitor for adult patients with relapsed or refractory acute myeloid leukemia (AML). IDHIFA was approved on 1 August 2017, along with a companion diagnostic, the RealTime IDH2 assay to detect IDH2 mutation [117,118]. In clinical trials, after a follow-up time of 6.6 months, 23% of patients taking 100 mg of Enasidenib once daily showed a complete response lasting a median of 8.2 months [118]. Another Agios inhibitor AG 120 (Ivosidenib) is currently in phase III clinical trials for metastatic cholangiocarcinoma (NCT02989857) and advanced hematologic malignancies with IDH1 mutations (NCT02074839). Additionally, Agios is also pursuing a phase I clinical trials for AG-881 (Vorasidenib) (Compound **6**), a brain penetrant and pan mutant IDH selective inhibitor in advanced solid tumors including gliomas with IDH1 and/or IDH2 mutation (NCT02481154) and for advanced hematologic malignancies (NCT02492737). Novartis has advanced IDH305 (Compound **22**) to phase 2 clinical trials for grade II and III gliomas (NCT02977689, temporarily withdrawn, pending safety trials). Other IDH inhibitors in clinical trials are Bayer’s BAY1436032 for IDH1 mutant solid tumors (NCT02746081), Forma Therapeutics’s FT-2102 (Compound **26**) for AML/MDS as a single agent (phase 1) and in combination with azacytidine (phase 2) are in clinical trials (NCT02719574).

### 5.2. Immunotherapy against the IDH1 Mutant Protein

IDH mutation is an early event in tumorigenesis and is seen uniformly distributed in all glioma cells making these mutations an ideal target for immunotherapy especially as a maintenance therapy for preventing recurrence of the tumors. Development of glioma-specific vaccine therapies has gained some attention to destroy tumor cells (Table 4). To date, none of phase 1 or 2 clinical trials of vaccine immunotherapies have specifically sought to target the IDH1 mutated epitope. No significant association between vaccine response and IDH1 R132H mutation status have been shown so far [143]. Three of these trials are listed below.

## 6. IDH Mutation-Induced Synthetic Lethality and Strategies for Synergistic Anticancer Efficacy

Besides the improved overall of survival of the patients, the altered metabolic and epigenetic profiles triggered by IDH mutations provide opportunities for synthetic lethality, where the cells expressing a particular oncogenic mutation exhibit heightened dependence on a sub-set of non-oncogenes for survival.

### 6.1. Are the IDH Inhibitors a Rational Means to Combat Cancers? Is There a Rethink Needed?

Recent studies that patients bearing IDH1/2 mutations in gliomas, cholangiocarcinomas, and to some extent the AML, experience a longer median survival than their wildtype counterparts, and they also show many favorable responses to radiotherapy and chemotherapy has prompted a new thinking on IDH1 mutations. Is there a benefit in disguise? How do we exploit it for more improved therapies? IDH1 mutations facilitate tumorigenesis through the oncometabolite by modifying the DNA, histone methylation and blocking the cell differentiation. Although the mutant IDH1 expression is an early event and drives the oncogenesis [144], some elegant studies have shown that the mutation rapidly assumes a passenger role in the malignant progression. Ablation of 2-HG failed to decrease histone methylation, adherent cell growth, or anchorage-independent growth in soft agar over a prolonged period in these experiments [145]. Therefore, curtailing the activity of the mutant enzyme may not be able to undo the chronic damage and epigenetic alteration in the genome already in place. Furthermore, there has been a disappointment on the lack of glioma patient responses following IDH1 inhibitor treatments [146]. These rationales prompted many research labs across the world, to exploit, rather than reverting, the IDH1/2 mutant phenotype for effective therapeutic strategies. With IDH mutations, researchers have found various non-oncogenes such as NAMPT, DNMT, glutaminase, BCl-2, and PARP offer a means to elicit synthetic lethality [98,136,137,147,148,149]. These examples are summarized in Table 5 and subsequent sections.

### 6.2. NAD^+^ Depletion

**Figure 5:**
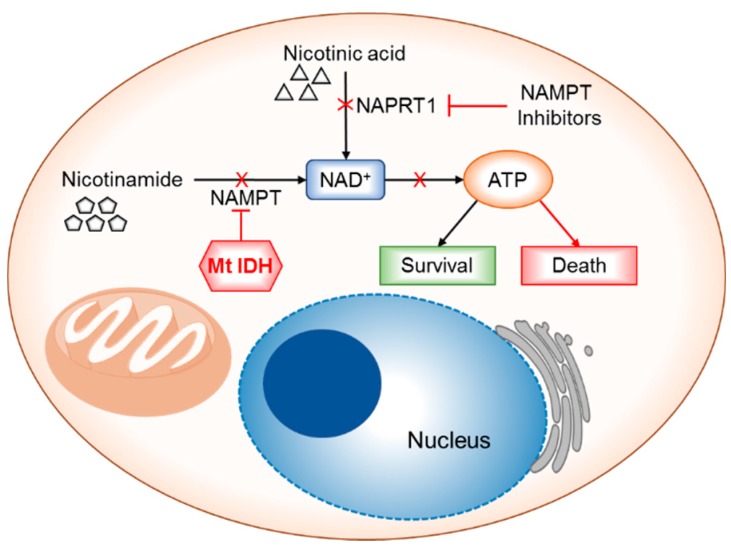
Schematic of the NAD^+^ salvage pathway in IDH mutant tumor cells as a strategy for energy deprivation and synthetic lethality. See text for description.

In attempts to identify the metabolic dependencies because of disrupted IDH function, Tateishi et al. found the profound vulnerability of IDH1 mutant glioma cells to the depletion of the NAD^+^ cofactor [136]. IDH mutations lead to a depletion of NAD^+^ pools, which is a known as key metabolite [151]. NAD^+^ levels in cells are maintained through a salvage pathway which has two branches that are regulated mainly by the salvage pathway enzymes, namely, the NAPRT1 (Nicotinate phosphoribosyltransferase 1) and NAMPT (Nicotinamide phosphoribosyltransferase). IDH1 mutations downregulate NAPRT, a rate-limiting enzyme in NAD^+^ salvage synthesis through the D-2HG mediated hypermethylation of NAPRT1 promoter CpG islands. The use of NAMPT inhibitors (synthetic lethality) caused a severe metabolic crisis inducing the activation of AMPK, and autophagy-mediated cell death in glioma cells and in mice harboring intracranial IDH mutant glioma xenografts. Several inhibitors of NAMPT are available and this strategy can be deemed as an effective and selective approach to kill IDH mutant cells as summarized in Figure 5.

### 6.3. DNA Hypermethylation

As discussed earlier, IDH mutations inhibit histone and DNA demethylases and are known to cause the malignant transformation. The DNMT1 inhibitor, decitabine has been demonstrated to effectively reverse the DNA hypermethylation and cellular differentiation induced by IDH mutations both in vitro and in vivo glioma models [137]. Others demonstrated that a long-term administration of low-dose azacytidine results in a reduction of DNA methylation at promoter sites and reduced cell proliferation in an anaplastic astrocytoma model [147]. Targeting the pathologic DNA hypermethylation in IDH mutant cells with DNA demethylating agents may represent a potential therapeutic approach and may need further clinical valuation in patients with IDH-mutant gliomas.

### 6.4. Metabolism

**Figure 6:**
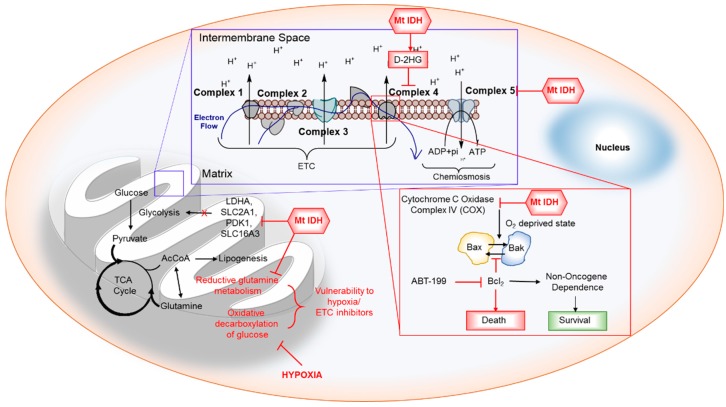
The IDH mutation induced metabolic changes. *Mt* IDH inhibits LDHA and glycolytic enzymes. It also inhibits reductive glutamine metabolism making IDH mutant cells vulnerable to ETC inhibitors and hypoxia. *Mt* IDH also inhibits Complex V of ETC and complex IV inhibition mediated by *Mt* IDH induced oxygen deprived state and they are dependent on BCl2 for their survival, whose inhibition induced synthetic lethality.

It is well known that IDH mutations induce a significant metabolic rewiring as shown in Figure 6. Chesnelong et al. demonstrated the *Mt* IDH silences the lactate dehydrogenase A (LDHA) through its promoter methylation. LDHA inactivation, in turn, downregulated several other glycolytic genes indicating the compromised glycolytic capacity of IDH mutant brain tumor stem cells (BTSCs) which may contribute to their slow growth and a better prognosis. IDH mutation-driven downregulation of LDHA indicated how different the metabolic profile of IDH1/2 mutant tumor is compared to other tumors [108]. In addition, D-2HG was earlier reported to impair the mitochondrial energy metabolism in rat tissues [152] and was recently reported to inhibit the ATP synthase and mTOR [153] indicating an altered overall metabolism as the reason for the slower growth of patient-derived IDH mutant cells [154]. Acetyl-coenzyme A (AcCoA), the central precursor for fatty acid synthesis, is primarily generated from glucose-derived pyruvate. However, highly proliferating cells with defective hypoxia signaling or impaired mitochondria utilize glutamine as the source for AcCoA. Glutamine contributes to the anabolic processes such as lipogenesis via the reductive glutamine pathway, in which glutamine is converted to acetyl-CoA via glutamate, α-KG, isocitrate, and citrate. The conversion of isocitrate to α-KG is critical and is catalyzed by *Wt* IDH under hypoxic conditions [155]. Studies of Reitman et al. revealed that IDH1 mutation causes cancer cells to switch toward reductive glutamine metabolism under hypoxia. IDH mutant cells contain decreased levels of glutamate and α-KG for which they depend on glutamine and have been found to be sensitive to glutaminase inhibitors [156,157]. Targeting the glutamine metabolism or using glutamine for targeted delivery of chemotherapeutic drugs can be a viable approach for selective cancer cell killing [148,158,159,160]. IDH mutations make cells sensitive to hypoxia and the electron transport chain inhibition in vitro. D-2HG also inhibits the ATP synthase s summarized in Table 6 and Figure 5. As the IDH mutant cells are deprived of the ATP, respiration and mTOR signaling and were found to be vulnerable to glucose limitation [153]. The IDH1 mutant cells and subcutaneous xenografts grew poorly within a hypoxic microenvironment, suggesting the amenability of metabolism in IDH mutant cells as a therapeutic target [161].

### 6.5. BCl-2 Dependence

A large-scale RNA interference (RNAi) screening to identify genes that are synthetic lethal to the IDH1 R132H mutation in AML revealed that IDH mutant cells are highly dependent on BCl-2. Chan et al. demonstrated that IDH1/2 mutant cells are more sensitive to ABT-199, a specific BCl2 inhibitor, than wildtype counterparts [149]. D-2HG inhibits complex IV, cytochrome C oxidase (COx). COx inhibition mimics a state of oxygen deprivation, which in turn activates Bax/Bak to trigger the mitochondrial outer membrane permeabilization (MOMP). However, BCl2 can maintain cell viability by antagonizing the Bax/Bak activation. As shown in Figure 6, treatment with ABT-199, a BH2 mimetic, disrupts the complex formation between BCl2 and Bax/Bak, unleashing the full activation of proapoptotic proteins and apoptosis [149]. Recently, it was reported that anaplastic astrocytoma specimens harboring IDH mutations displayed lower levels of Mcl-1. Addition of D-2HG to GBM cell cultures reduced the Mcl-1 expression and sensitized the glioblastomas to Bcl-XL inhibition both in vitro and in intracranial xenografts [162].

### 6.6. Defective Homologous Recombination, Increased Sensitivity to PARP Inhibitors and Altered DNA Damage Responses (DDR) in IDH-Mutated Cancers

**Figure 7:**
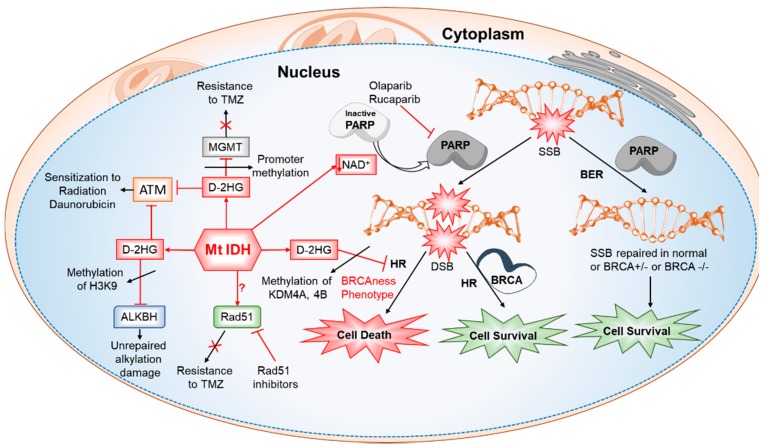
Mutations in IDH altered various components of DNA damage repair mechanisms. D-2HG mediated histone methylation inhibits ALKBH. D-2HG inhibits ATM, the DNA damage sensor. Decreased MGMT expression is known to happen during IDH mutations owing to increased promoter methylation and is reported to be the reason for increased sensitivity to TMZ therapy in those patients. Depleted levels of NAD^+^ in IDH mutant cells leads to improper activation of PARP. D-2HG mediated histone methylation inhibits HR creating a BRCAness phenotype making the cells vulnerable to PARP inhibitors.

DNA damage response machinery works like a double-edged sword. Double strand breaks are the potentially lethal lesions [163] and homologous recombination (HR) and non-homologous end joining (NHEJ) are the two pathways involved in their repair [164]. ATM plays a key role in recruiting DDR machinery in HR pathway. Mutations or inactivation of DDR proteins in stem cells especially leads to carcinogenesis. Conversely, overexpression of these proteins in cancers leads to chemotherapy resistance. Clinical studies have suggested a link between IDH1/2 mutations and enhanced chemo- or radiation-sensitivity, indicating the possible DDR defects can enhance cells susceptibility to DNA damaging agents. In this context, Sulkowski et al. [146] analyzed the ability of IDH mutant cells to recover from the DNA damage and found the IDH1/2 mutant cells are deficient in homologous repair of double-strand breaks (DSBs). Further screening of IDH mutant cells for their sensitivity against various DDR inhibitors revealed their significant sensitivity to PARP inhibitors. Other studies confirmed that IDH1/2 mutation induced HR defects to occur by D-2HG mediated inhibition of lysine demethylases, KDM4A and KDM4B, which mimic the BRCAness phenotype and this renders IDH mutant cells sensitive to PARP inhibition. In addition, treatment with IDH mutant-specific inhibitors reverted the observed HR defects and eliminated the associated PARP inhibitor sensitivity. NAD^+^ depletion associated with IDH mutations was further linked to a deficient base excision repair pathway. Lu et al. recently reported on synergistic effects between TMZ and Olaparib in IDH mutant patients providing the possibility to achieve improved cytotoxic effects with minimal use alkylating agents to reduce bone marrow cytotoxicity [165]. Further, the mutant IDH1 downregulated the DNA damage sensor ATM via methylation of H3K9 in a TET2 independent manner, leading to an impaired DDR and increased sensitivity to DNA damaging agents and in turn to an attenuated self-renewal of hematopoietic stem cells (HSCs) in AML [98] (Figure 7).

### 6.7. Oxidative Stress: NADPH Depletion and Generation of Reactive Oxygen Species (ROS)

**Figure 8:**
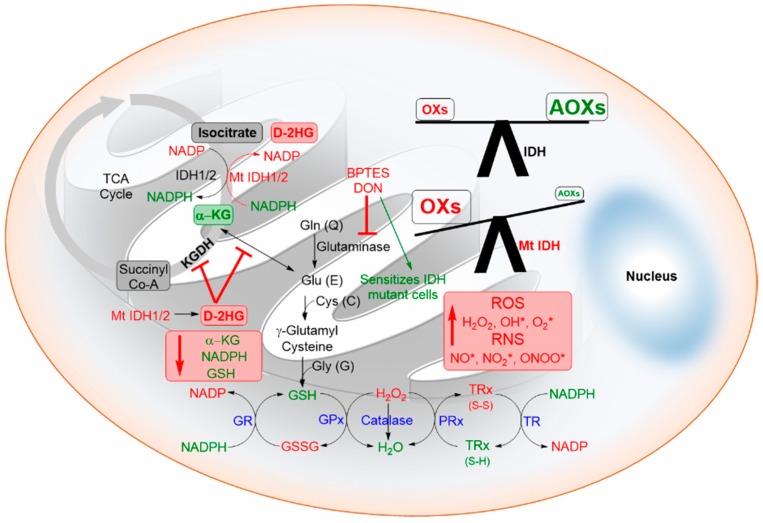
Redox imbalance induced by IDH mutations. IDH mutations lead to depleted levels of crucial antioxidants such as α-KG, NADPH, and GSH. D-2HG also inhibits the ROS sensor α-KGDH. The oxidative stress can inactivate pivotal regulators such as the p53, AP1, and NRF2 transcription factors. Furthermore, the antioxidant thiol metabolic/enzymic systems (glutathione, peroxiredoxin, thioredoxin, glutaredoxin) are also finely altered during redox alterations. Many of these cellular pathways are likely to provide fresh strategies for drug discovery for sensitizing the IDH-mutant cancer cells. BPTES and DON are inhibitors of glutaminase. (Oxs, oxidants; Aoxs, antioxidants, RNS, reactive nitrogen species, TRx, Thioredoxin; GR, Glutathione reductase; GPx, Glutathione peroxidase; PRx, Peroxiredoxin; TR, Thioredoxin reductase).

The tumor microenvironment is characterized by enhanced cellular stresses due to oxidative, replicative, metabolic, proteotoxic and DNA damage. Cancer cells adapt to this stress phenotype for their survival by altering their antioxidants and modulating several non-oncogenes that normally do not perform such vital functions [166]. Though elevated ROS in cancer cells maintain the cancer phenotype, they interact with various biomolecules (proteins, lipids, DNA) producing stable and highly reactive aldehydes which further intensify the genetic instability [167]. It is assumed that targeting such non-oncogene dependencies of cancer cells may result in synthetic lethal interaction [168] and selective death of cancer cells [169,170]. Shi et al. postulated that the depleted NADPH and GSH levels cause redox imbalance resulting in elevated ROS, which further induces chemo/radiation sensitivity [6,44]. The total NADPH production capacity in glioblastoma was provided for 65% by IDH activity and the occurrence of IDH1R132 mutation reduced this capacity by 38% [171]. Molenaar et al. recently reported that better prognosis of patients with established IDH mutations in glioma or cholangiocarcinoma may be related to increased oxidative stress in these tumors due to lower NADPH production capacity [44]. The IDH induced changes to the redox balance are summarized in Figure 8.

## 7. Can We Learn from the Oncometabolite Pathology and Turn the Tide against the Primary Glioblastomas Which Do Not Bear IDH1 Mutations?

IDH1/2 mutations prevail (>85% of cases) in diffuse gliomas (grades II and III). Regarding glioblastoma, they are encountered only in 5% of them, in the so-called secondary glioblastoma that derives from diffuse gliomas. The vast majority of glioblastoma is, therefore, IDH wildtype with variable predominant mutations and other genomic alterations. The glioblastomas, both in pediatric and adult patients remain the most lethal malignancies. Prognosis remains dismal and median overall survival rarely exceeds 12 months. Radiation and adjuvant temozolomide have remained the mainstay of GBM treatment. There is an urgent need for new and innovative treatment modalities for this tumor type. Having known the consistent epigenetic modifications engineered by D-2HG through its constant exposure in lower grade gliomas and an overall better therapeutic response in patients thereof, a provocative, but compelling question is whether the oncometabolite per se or better analogs of D-2HG would be useful in imparting therapy-relevant genomic modifications in glioblastomas? Supportive of this intriguing proposal is that D-2HG and its cell-penetrating esters do induce de novo histone methylations and DNA methylations in non-IDH mutant cancer cells [91,100,161]. Another possibility is that powerful alpha-KG derivatives that can replace the natural metabolite in epigenetic dioxygenase reactions may serve as potent drugs for GBMs. Such drugs, in an intermittent therapeutic setting, may still be tolerable without adverse effects, and additionally are expected to increase the tumor redox stress, which in turn may enhance the drug sensitivity. We believe these approaches are relevant and merit further investigation. Notwithstanding the knowledge already gained, it is imperative that we should continue to dissect the finer molecular details of oncometabolite—induced pathophysiology to derive therapeutic benefits.

## Figures and Tables

**Table 1:** Summary of mutations with respect to nucleotide and amino acid changes in IDH1/2.

Gene	Nucleotide Change	Amino Acid	References
IDH1	G395A	R132H	[12,16,17,18,19]
	C394T	R132C	[12,16,17,18,19]
	C394G	R132G	[12,16,17,18,19]
	C394A	R132S	[12,16,17,18,19]
	G395T	R132L	[12,16,17,18,19]
	G299A	R100Q	[16,20]
IDH2	G515A	R172K	[16,18,20,21]
	G515T	R172M	[18,21]
	A514T	R172W	[18,21]
	G516C	R172S	[18,21]
	G419A	R140Q	[18,20,21]
	G419T	R140L	[18,20,21]
	C418T	R140W	[18,20,21]
	C418G	R140G	[18,20,21]

**Table 2:** Summary of Prevalence of IDH1/2 Mutations.

Tumor Type	Total # of Patients	IDH1 Mutations	IDH2 Mutations	Reference
Diffuse astrocytoma (II)	30	25 (83.3%)	2 (6.7%)	[12]
Anaplastic astrocytoma (III)	52	36 (69.2%)	2 (3.8%)	[12]
Secondary GBM (IV)	13	11 (84.6%)	0	[12]
Primary adult GBM (IV)	123	6 (4.8%)	0	[12]
Primary pediatric GBM (IV)	15	0	0	[12]
Oligodendroglioma (II)	51	41 (80.4%)	2 (3.9%)	[12]
Anaplastic oligodendroglioma (III)	36	31 (86.1%)	3 (8.3%)	[12]
Oligoastrocytoma (II)	3	3 (100%)	0	[12]
Anaplastic oligoastrocytoma (III)	7	7 (100%)	0	[12]
Ependymoma (II) and Medulloblastoma (IV)	85	0	0	[12]
AML	805	61 (7.6%)	129 (16%)	[31]
	145	50 (34.5%)	50 (34.5%)	[32]
Central Chondrosarcoma (II and III)	39	18 (46.2%)	5 (12.8%)	[19]
Central cartilaginous tumors	75	38 (50.7%)	1 (1.3%)	[19]
Dedifferentiated Chondrosarcoma	23	12 (52.2%)	1 (4.3%)	[19]

**Table 3:** Structures and description of mutant-specific inhibitors of IDH1/that are in various stages of drug development. Chemical structures from various sources were redrawn using Chem Draw (Perkin Elmer, Waltham, MA, USA).

Compound	Inhibitor	Structure	Comments	Target	References
**1**	IDHifa^®^ (AG-221 Enasidenib)	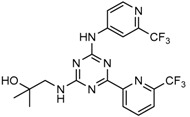	FDA approved for AML Orally available Potent inhibitor of *Mt* IDH2	IDH2-R140Q IDH2-R172H	Agios Celgene [116,117,118]
**2**	AGI-5198	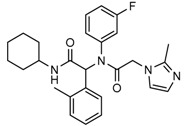	IC_50_ > 20 μM against U87R132H or HT1080	IDH1-R132H IDH1-R132C	Agios [119,120]
**3**	IDH-C227	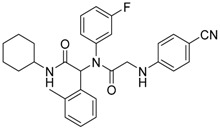	IC_50_ < 0.1 μM against HT1080 and 0.25 μM against U87MG	IDH1-R132H	Agios US20130035329A1
**4**	AGI-6780	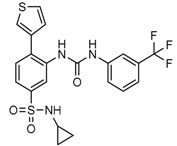	Allosteric Binds to dimer interface K_i_ 23 nM against IDH2 R140Q vs. 11 μM IDH1 R132H	IDH2-R140Q	Agios [116,121]
**5**	AG-120 (Ivosidenib)	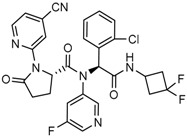	Orally available Reversible inhibitor	IDH1-R132H IDH1-R132C	Agios [122]
**6**	AG-881 (Vorasidenib)	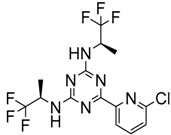	BBB penetrable Orally available	Pan-IDH mutants	Agios NCT02481154
**7**	ML309	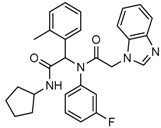	K_i_ 96 nM Impermeable to BBB	IDH1-R132H IDH1-R132C	[123]
**8**	EXEL-9324	Not revealed	K_i_ = 298 nM against *Wt*/*Mt*	IDH1 *Wt*/*Mt*	[27]
**9**	VVS	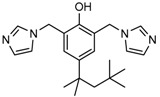	Allosteric Binds to the interface IC_50_ 81.5 nM on HEK-293 R132H	IDH1-R13H IDH1-R132C	[124]
Tetrahydropyrazolopyridine		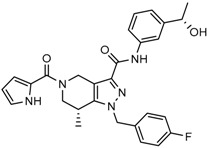	Allosteric K_i_ 3–5 nM against IDH1 R132H/C/G Binds to each subunit rather than at the interface of dimer Reduced or reversed the hypermethylation of histones/DNA in HT1080 cells, EC_50_ 85 nM	IDH1-R132H	[125]
**10**	GSK321				
**11**	GSK990	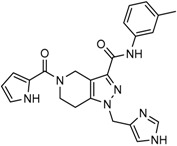			
**12**	FX-03	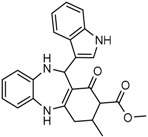	IC_50_ 55 and 65 μM against mutant IDH expressing cells	*Mt* IDH	[126]
**13**	1-Hydroxypyridin-2-one	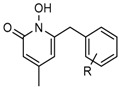	K_i_ 120–140 nM against IDH1 R132C/H IC_50_ 260 nM on mutant IDH1 glioma cells	IDH1-R132H IDH1-R132C	[127]
**14**	Compound **3**	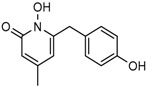	K_i_ 120 nM EC_50_ 2.4 μM Binds to the ligand binding site rather than the catalytic active site → selectivity	IDH1-R132H IDH1-R132C	[128]
**15**	SYC-435	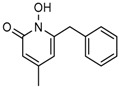			
**16**	Compound **20a**	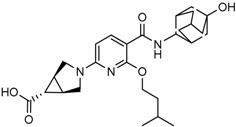	Allosteric EC_50_ to inhibit D-2HG production 1.9 μM and to basal levels at ≥10 μM	IDH1	[129]
2-Thiohydantoin Compounds		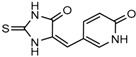	K_i_ 420 nM against IDH1 R132H	IDH1-R132H	[130]
**17**	Compound **4**	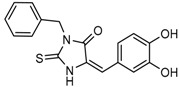	K_i_ 4.7 μM		
**18**	Compound **16**	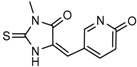	K_i_ 0.4–0.75 μM against R132H Allosteric Binds to protein deep in the cleft in between dimer		
**19**	Compound **18**	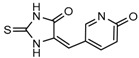			
**20**	IDH889	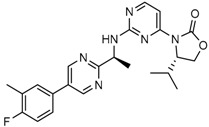	IC_50_ 0.02 μM IDH1 R132H Allosteric BBB penetrable	IDH1-R132H	Novartis [131]
**21**	IDH125	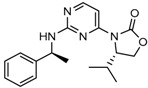	IC_50_ 0.22 μM IDH1 R132H Allosteric BBB penetrable		
**22**	IDH305	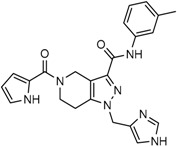	Potent, orally available, EC_50_ 24 nM	IDH1-R132H	Novartis [132]
**23**	3-pyrimidin-4-yl-oxazolidin-2-one (Novartis-556)	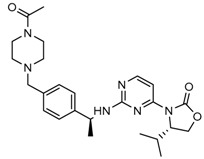	Mutant IDH1 inhibitor K_i_ 72 nM	IDH1	Novartis US9688672B2 [133]
**24**	Bay1436032	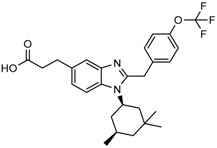	Orally available Prolonged survival in two independent PDX *Mt* IDH1 AML mouse model	Pan-mutant IDH1	[134,135]
**25**	BRD2879	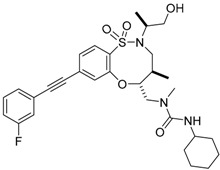	K_i_ 50 nM against IDH1 R132H	IDH1-R132H	[136]
**26**	FT-2102	Undisclosed	In phase 1 clinical trials AML and MDS	IDH1 mutant	Forma Therapeutics NCT02719574
**27**	Clomifene	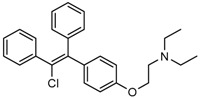	Virtual screening revealed Non-competitive inhibitor Effective in both in vitro and in vivo	IDH1-R132H	[137]

AML—acute myelogenous leukemia; IDH—isocitrate dehydrogenase; BBB—blood brain-barrier; PDX—patient derived xenograft, MDS—myelodysplastic syndrome.

**Table 4:** Immunotherapeutic options in clinical trials against the mutant IDH.

Vaccine	Mechanism of Action	Indication	Clinical Trials
IDH1 R132H dendritic vaccine	Immunotherapy	IDH1 R132H glioma	NCT02771301
IDH1 R132H peptide vaccine	Immunotherapy	IDH1 R132H glioma	NCT02454634
PEPIDH1M vaccine	Immunotherapy	Progressive of recurrent grade II gliomas	NCT02193347

**Table 5:** Summary of opportunities through which IDH mutations can be exploited for increased antitumor efficacy through synthetic lethality.

Drug	Mechanism of Action	Indication	Ref.
FK866GMX1778	NAMPT inhibitors	IDH mutant cells are vulnerable to NAD^+^ depletion	[136]
Decitabine	DNMT1 inhibitors	Hypermethylation induced by IDH mutations can be reversed	[137,147]
Azacytidine			
Olaparib, MK-4827, Rucaparib, BMN-673	PARP inhibitors	D-2HG suppresses HR inducing BRCAness phenotype and induces PARP inhibitor sensitivity	[148]
BPTES CB-839	Glutaminase Inhibitor	Reductive glutamine metabolism is seen in IDH mutant cells. Glutamine serves as a source for α-KG and its inhibition slows the growth of IDH mutant cells	[148,150] NCT02071862
ABT-199	BCl-2 inhibitors	IDH mutant cells have non-oncogene dependence on BCl-2, whose inhibition sensitizes IDH mutant cells	[149]
Daunorubicin, IR	DNA damaging agents	D-2HG inhibits ATM and IDH mutant cells become sensitive to DNA damaging agents	[98]

Abbreviations: NAMPT, Nicotinamide phosphoribosyl transferase; DNMT, DNA methyltransferase; BRCA, Breast cancer-associated genes 1/2; IR, Ionizing radiation; HR, Homologous recombination; ATM, Ataxia-telangiectasia mutated.

**Table 6:** Interplay between IDH mutant status and hypoxia in survival/growth of cells.

Normal growth conditions	*Wt* IDH	Regular glucose metabolism through oxidative decarboxylation in mitochondria (Both *Wt* IDH or *Mt* IDH (heterozygous)	Growth supported
	*Mt* IDH		
Hypoxia/ETC inhibition	*Wt* IDH	Reduced glucose flux but Glutamine metabolism compensates for *Wt* IDH	Growth supported
	*Mt* IDH	Oxidative glucose metabolism is inhibited. However, *Mt* IDH cannot induce reductive glutamine metabolism	No growth or compromised growth
Normal growth conditions	*Wt* IDH	Regular glucose metabolism through oxidative decarboxylation in mitochondria (Both *Wt* IDH or *Mt* IDH (heterozygous)	Growth supported
	*Mt* IDH		
Hypoxia/ETC inhibition	*Wt* IDH	Reduced glucose flux but Glutamine metabolism compensates for *Wt* IDH	Growth supported
	*Mt* IDH	Oxidative glucose metabolism is inhibited. However, *Mt* IDH cannot induce reductive glutamine metabolism	No growth or compromised growth

Abbreviations: ETC, Electron Transport Chain.
